# Divergent associations of TyG index and HOMA-IR with cardiovascular and cerebrovascular mortality in non-diabetic elderly: a competing risk analysis of NHANES 2003‒2020

**DOI:** 10.1016/j.clinsp.2026.101089

**Published:** 2026-09-06

**Authors:** Xintian Xu, Xiaomei Ye, Chen Xiao, Yaozhuo Cai, Hao Chen, Xueli Cai, Jingping Sun

**Affiliations:** a Department of Neurology, The Fifth Affiliated Hospital of Wenzhou Medical University (Lishui Central Hospital), Lishui, Zhejiang, China; b Department of Neurology, Lishui People’s Hospital, Lishui, Zhejiang, China; c Lishui Clinical Research Center for Neurological Diseases, Lishui, Zhejiang, China

**Keywords:** Insulin resistance, Triglyceride-glucose index, Cardiovascular and cerebrovascular diseases, Competing risk, NHANES

## Abstract

**Background and purpose:**

While Triglyceride-Glucose (TyG) index links to Cardiovascular and Cerebrovascular Diseases (CVDs), prior studies neglected competing risks and direct comparisons with Homeostatic Model Assessment for Insulin Resistance (HOMA-IR). We evaluated and compared their associations with CVDs mortality under competing risks among non-diabetic adults aged ≥ 45-years.

**Methods:**

Using NHANES data (2003‒2020), we analyzed 8735 non-diabetic participants. TyG and HOMA-IR were calculated for each participant. Mortality outcomes included all-cause, CVDs, cardiac and cerebrovascular disease. We used Cox proportional hazards regression with C-statistic comparison, along with restricted cubic splines to examine nonlinear associations. To address competing risks, Fine‑Gray models were used to evaluate cause‑specific mortality, followed by a sensitivity analysis excluding participants with baseline CVDs.

**Results:**

Among 1,804 total deaths (599 CVDs), an elevated TyG index showed linear positive associations with risks of all-cause mortality (Model 3, adjusted Hazard Ratios [HR = 1.19], p = 0.005), CVDs mortality (HR = 1.31, p = 0.02), and cerebrovascular mortality (HR = 1.92, p < 0.001). For HOMA-IR, continuous-variable analysis revealed significant linear associations with several mortality outcomes, whereas tertile and RCS analyses suggested more complex, nonlinear relationships. C-statistic comparisons showed no significant improvement in predictive discrimination with either index beyond traditional risk factors. However, in competing risk models, higher TyG remained associated with increased CVDs mortality, while HOMA-IR was associated with a lower subdistribution hazard.

**Conclusion:**

Among non-diabetic older adults, the TyG index demonstrated a linear association with CVDs mortality, while HOMA-IR showed a more complex J-shaped pattern. Future research could explore interventions targeting the TyG index to potentially improve survival in the elderly.

## Highlights

• In non-diabetic elderly, TyG index linearly, HOMA-IR J-shapedly, linked to CVDs death.
• First competing risk model reveals HOMA-IR’s protection reflects higher non-CVDs death.
• TyG and HOMA-IR lack incremental mortality prediction beyond CVDs risk factors.
• TyG independently predicts stroke death in healthy elders, aiding primary prevention.

## Introduction

Cardiovascular and Cerebrovascular Diseases (CVDs) remained the leading cause of global age-standardized mortality until 2021, imposing a substantial public health burden.^1^ In 2020 alone, cardiac disease and stroke caused more deaths than cancer and chronic respiratory diseases combined.^2^ Consequently, identifying modifiable risk factors for early intervention is critical to reduce CVDs mortality. The pathophysiological basis of CVDs is strongly linked with metabolic disorders, particularly Insulin Resistance (IR),^3^ characterized as a diminished sensitivity or responsiveness to the metabolic actions of insulin.^4^ The most widely utilized surrogate markers for IR are Homeostatic Model Assessment for Insulin Resistance (HOMA-IR) and the Triglyceride-Glucose (TyG) index.^5^^,^^6^

Studies have indicated that the TyG index as a superior predictor of IR compared to HOMA-IR.^7^ TyG demonstrates significantly stronger correlations with arterial stiffness^8^ and atherosclerotic plaque burden,^9^ indicating enhanced cardiovascular risk stratification capacity.^10^ Consequently, compared to the limited evidence supporting HOMA-IR as an independent predictor of cardiovascular death,^11^^,^^12^ extensive research has focused on elucidating TyG’s associations with incident cardiovascular disease and long-term prognosis. Elevated TyG index increases the risk of impaired cardiovascular fitness in non-diabetic young populations,^13^ and shows significant associations with 1-year major adverse cardiovascular events risk in coronary artery disease patients.^14^^,^^15^ However, the association between the TyG index and cardiovascular mortality is not consistent across populations with different glucose-metabolic statuses and age groups.^16^^,^^17^

Current evidence on TyG’s association with cerebrovascular mortality, and its predictive capacity for CVDs mortality relative to HOMA-IR remains limited. Critically, prior investigations have inadequately considered competing mortality risks, including cancer and infections. Therefore, we aim to investigate the TyG’s relationship with CVDs mortality under competing risks in non-diabetic older adults, with direct comparison to HOMA-IR.

## Materials and methods

### Study design and population

This study utilized data from the National Health and Nutrition Examination Survey (NHANES) database spanning 2003–2020. NHANES represents an ongoing cross-sectional survey employing a complex, multistage probability sampling design to obtain nationally representative data on the health and nutritional status of U.S. adults and children. The NHANES study protocol received approval from the National Center for Health Statistics (NCHS) Research Ethics Review Board, and all participants provided written informed consent. Consequently, no additional ethical review or informed consent was required for our secondary analysis. The datasets generated and analyzed in this study are readily accessible on the website (https://www.cdc.gov/nchs/nhanes/index.html).

The initial cohort included 28,029 participants after screening individuals aged ≥ 45 years. After excluding individuals with diabetes (n = 5,570), missing preexisting diabetes data (n = 22), incomplete TyG or HOMA-IR measurements (n = 12,790), and those lacking outcome data (n = 912), the final analytical sample comprised 8,735 participants.

### Assessment of TyG and HOMA-IR

This study focused on two primary biomarkers of IR: the TyG index and HOMA-IR index. Venous blood samples were collected from participants after an overnight fast for measurement of plasma triglycerides, fasting glucose, and fasting insulin. Calculations were performed according to the following validated formulae:

(1) TyGindex=Ln[Fastingtriglycerides(mg/dL)×Fastingglucose(mg/dL)/2];^18^
(2) HOMA−IR=[Fastingglucose(mmol/L)×Fastinginsulin(μU/mL)]/22.5.^19^

### Assessment of mortality

The primary mortality endpoints included: 1) All-cause mortality; 2) CVDs mortality; 3) Cardiac mortality; and 4) Cerebrovascular mortality.

Mortality data were ascertained using the NHANES Public-Use Linked Mortality File (through December 31, 2019), which employs probabilistic matching algorithms administered by the NCHS to link participant records with the National Death Index (NDI). Cause-specific mortality was classified according to the International Classification of Diseases, Tenth Revision (ICD-10) codes. CVDs encompass cardiac diseases (including rheumatic heart disease, hypertensive heart disease, ischemic heart disease, acute myocardial infarction, pericardial diseases, acute myocarditis, and heart failure; ICD-10 codes I00-I09, I11, I13, I20-I51) and cerebrovascular diseases (ICD-10 codes I60-I69).

Cause-specific mortality quantifies the death risk for a particular disease when competing events coexist. Participants were stratified into three states: 1) Cause-specific death (target disease), 2) Competing-cause death, or 3) Censored.

### Assessment of covariates

We extracted comprehensive demographic and health-related data from NHANES. Demographic characteristics included age, sex (female/male), and race (Mexican American / Other Hispanic / Non-Hispanic Black / Non-Hispanic White / Other Race including multi-racial), Body Mass Index (BMI), waist circumference, married status(married/divorced/never married), and Poverty-Income Ratio (PIR), educational attainment (less than 9^th^ grade / 9‒11^th^ grade / high school graduate or equivalent / some college or AA degree / college or above), smoking status (yes/no), and alcohol consumption patterns (yes/no). Preexisting medical conditions contained self-reported histories of hypertension, dyslipidemia, CVDs, and malignancy. Laboratory measurements featured fasting plasma glucose, fasting insulin, total cholesterol, triglycerides, Low-Density Lipoprotein Cholesterol (LDL-C), and High-Density Lipoprotein Cholesterol (HDL-C).

### Statistical analysis

All statistical analyses were conducted in accordance with CDC guidelines. Due to the complex sampling design of NHANES, sample weights were applied to combine data from multiple survey cycles for statistical analysis. Following NHANES analytic guidelines for combining multiple survey cycles, we constructed pooled sampling weights by dividing the original 2-year interview weights (wtint2yr) by the number of cycles combined (9 cycles: 2003‒2020). These pooled weights account for the complex multistage probability sampling design and were applied in all analyses using the R survey package to obtain nationally representative estimates with appropriate standard errors. Participants were stratified into three groups (T1‒T3) based on tertiles of the TyG index and HOMA-IR index, respectively. For group difference analysis, considering the variability and uncertainty introduced by the NHANES sampling process, continuous variables are presented as mean ± Standard Error (SE), while categorical variables are expressed as weighted percentages (%). We utilized multivariable Cox proportional hazards regression models to estimate the Hazard Ratios (HRs) and 95% Confidence Intervals (95% CIs) for the associations of the TyG/HOMA-IR index with all-cause mortality, CVDs mortality, cardiac mortality and cerebrovascular mortality. There were three models to control for confounding factors. Model 1 was unadjusted. Model 2 was adjusted for age, sex, and race. Model 3 was adjusted for age, sex, race, smoking, alcohol, BMI, hypertension, dyslipidemia, prevalent cardiovascular diseases and stroke. Missing data for covariates (< 30% for all variables) were handled using Multiple Imputation by Chained Equations (MICE) with 5 imputed datasets. Imputation models included all covariates and outcome indicators. Convergence was assessed by examining trace plots of means and standard deviations across iterations. Estimates from imputed datasets were combined using Rubin's rules. Restricted Cubic Splines (RCS) with three knots anchored at the 10^th^, 50^th^, and 90^th^ percentiles of TyG/HOMA-IR distributions were implemented to characterize potential nonlinear relationships with clinical outcomes. To compare the predictive discrimination of models incorporating the TyG index versus HOMA-IR, we calculated Harrell’s C-statistic for each fully adjusted model (Model 3) across all mortality outcomes. To account for competing risks, we employed Fine‑Gray subdistribution hazard models to assess the associations of the TyG index and HOMA‑IR with cause‑specific mortality. To examine the robustness of our primary findings from the competing risk analysis, we performed a sensitivity analysis after excluding all participants with pre-existing CVDs at baseline.

All statistical analyses were performed using R software version 4.5.2. A two-tailed p < 0.05 was considered statistically significant.

## Results

### Baseline characteristics

Table 1 present the baseline characteristics of the study participants stratified by tertiles of the TyG and HOMA-IR indices. The study included 8,735 non-diabetic participants with an average age of 59.96-years, 46.88% of whom were male. The tertile ranges for the TyG index were T1 (≤ 8.38), T2 (8.38–8.73), and T3 (≥ 8.73). Similarly, the tertile ranges for the HOMA-IR index were T1 (≤ 1.47), T2 (1.47–2.84), and T3 (≥ 2.84). Both indices revealed a highly consistent risk-stratification pattern: with increasing TyG or HOMA-IR, participants showed a marked clustering of metabolic risk factors and a lower socioeconomic status. Specifically, higher index values were associated with elevated BMI, waist circumference, fasting plasma glucose, triglycerides, total cholesterol, and fasting insulin, along with significantly lower HDL-C (all trend p < 0.001). The prevalence of hypertension, dyslipidemia, and pre-existing CVDs was also significantly greater (all p < 0.05). In addition, groups with higher index values had a higher proportion of males but lower family PIR and lower educational attainment (all p < 0.001). However, the proportion of current drinkers only decreased with higher HOMA-IR (p = 0.001). During follow-up, 1,804 all-cause deaths occurred, including 599 CVDs deaths (476 cardiac disease, 123 cerebrovascular disease).

**Table 1:** Baseline characteristics according to tertiles of the TyG and HOMA-IR index.

Variable	Total (n = 8735)	Tertiles of the TyG index				Tertiles of the HOMA-IR index			
		T1 (n = 2912)	T2 (n = 2911)	T3 (n = 2912)	p	T1 (n = 2912)	T2 (n = 2911)	T3 (n = 2912)	p
**Demographics**									
Age (y), mean (SE)	59.96 (0.18)	59.46 (0.31)	60.18 (0.26)	60.25 (0.25)	0.08	59.51 (0.30)	60.49 (0.26)	59.94 (0.24)	0.02
Sex (male), n (%)	4256 (46.88)	1313 (40.93)	1428 (48.27)	1515 (51.68)	<0.001	1386 (42.96)	1406 (46.38)	1464 (51.88)	<0.001
Non-Hispanic white, n (%)	4385 (76.34)	1353 (76.43)	1469 (76.43)	1563 (78.28)	<0.001	1614 (79.59)	1452 (75.93)	1319 (73.02)	<0.001
BMI (kg/m2), mean (SE)	28.68 (0.11)	26.75 (0.15)	28.69 (0.15)	30.70 (0.17)	<0.001	25.11 (0.11)	28.53 (0.13)	32.95 (0.19)	<0.001
Waist circumference (cm), mean (SE)	100.11 (0.26)	94.58 (0.40)	100.15 (0.36)	105.83 (0.42)	<0.001	90.99 (0.30)	100.01 (0.32)	110.69 (0.41)	<0.001
Married, n (%)	5129 (65.21)	1658 (65.40)	1686 (63.88)	1785 (66.34)	0.74	1700 (66.63)	1727 (66.08)	1702 (62.68)	0.03
PIR, mean (SE)	3.26 (0.04)	3.41 (0.05)	3.22 (0.05)	3.15 (0.05)	<0.001	3.38 (0.06)	3.25 (0.05)	3.14 (0.05)	<0.001
College graduate or above, n (%)	1997 (30.43)	835 (38.69)	634 (28.96)	528 (23.27)	<0.001	782 (36.52)	666 (29.12)	549 (24.76)	<0.001
Current smoker, n (%)	4431 (50.57)	1364 (46.08)	1484 (50.31)	1583 (55.54)	<0.001	1511 (49.84)	1445 (50.53)	1475 (51.46)	0.71
Current drinker, n (%)	6090 (75.50)	2062 (77.09)	2036 (75.41)	1992 (73.94)	0.13	2118 (78.08)	2026 (75.34)	1946 (72.70)	0.001
**Medical history**									
Hypertension, n (%)	4111 (42.63)	1239 (34.30)	1322 (42.29)	1550 (51.67)	<0.001	1072 (30.04)	1364 (43.71)	1675 (56.01)	<0.001
Dyslipidemia, n (%)	4106 (47.61)	1054 (36.33)	1390 (47.47)	1662 (59.56)	<0.001	1187 (40.47)	1424 (49.37)	1495 (54.04)	<0.001
CVDs, n (%)	1175 (11.21)	355 (10.03)	382 (10.74)	438 (12.92)	0.02	345 (8.97)	386 (11.31)	444 (13.69)	<0.001
Self-reported cancer, n (%)	1239 (15.38)	410 (15.01)	400 (14.85)	429 (16.30)	0.54	412 (14.55)	428 (15.84)	399 (15.87)	0.34
**Laboratory examination**									
FPG (mmoL/L), mean (SE)	5.76 (0.02)	5.42 (0.01)	5.71 (0.02)	6.17 (0.03)	<0.001	5.34 (0.01)	5.70 (0.02)	6.32 (0.03)	<0.001
Insulin (pmoL/L), mean (SE)	69.89 (0.87)	48.05 (1.01)	65.95 (1.04)	96.71 (1.99)	<0.001	28.13 (0.24)	57.08 (0.30)	131.04 (1.78)	<0.001
TC (mmoL/L), mean (SE)	5.25 (0.02)	4.94 (0.02)	5.24 (0.03)	5.58 (0.03)	<0.001	5.30 (0.03)	5.32 (0.02)	5.13 (0.03)	<0.001
TG (mmoL/L), mean (SE)	1.46 (0.01)	0.74 (0.01)	1.25 (0.01)	2.42 (0.03)	<0.001	1.15 (0.02)	1.49 (0.03)	1.78 (0.03)	<0.001
HDL-C (mmoL/L), mean (SE)	1.48 (0.01)	1.73 (0.01)	1.46 (0.01)	1.22 (0.01)	<0.001	1.67 (0.01)	1.46 (0.01)	1.26 (0.01)	<0.001
LDL-C (mmoL/L), mean (SE)	3.11 (0.02)	2.87 (0.02)	3.21 (0.02)	3.25 (0.02)	<0.001	3.10 (0.02)	3.18 (0.02)	3.05 (0.03)	<0.001

TyG, Triglyceride Glucose Index; HOMA-IR, Homeostatic Model Assessment of Insulin resistance; BMI, Body Mass Index; PIR, Poverty Income Ratio; CVDs, Cardiovascular and Cerebrovascular Diseases; FPG, Fasting Plasma Glucose; TC, Total Cholesterol; TG, Triglyceride; HDL-C, High-Density Lipoprotein Cholesterol; LDL-C, Low-Density Lipoprotein Cholesterol; SE, Standard Error. Percentages are weighted to account for NHANES complex survey design; raw Ns are unweighted counts. Weighted percentages may not correspond directly to raw Ns due to differential sampling probabilities.

### Association of the TyG index and HOMA-IR with all-cause, CVDs, cardiac and cerebrovascular mortality

**Fig. 1:**
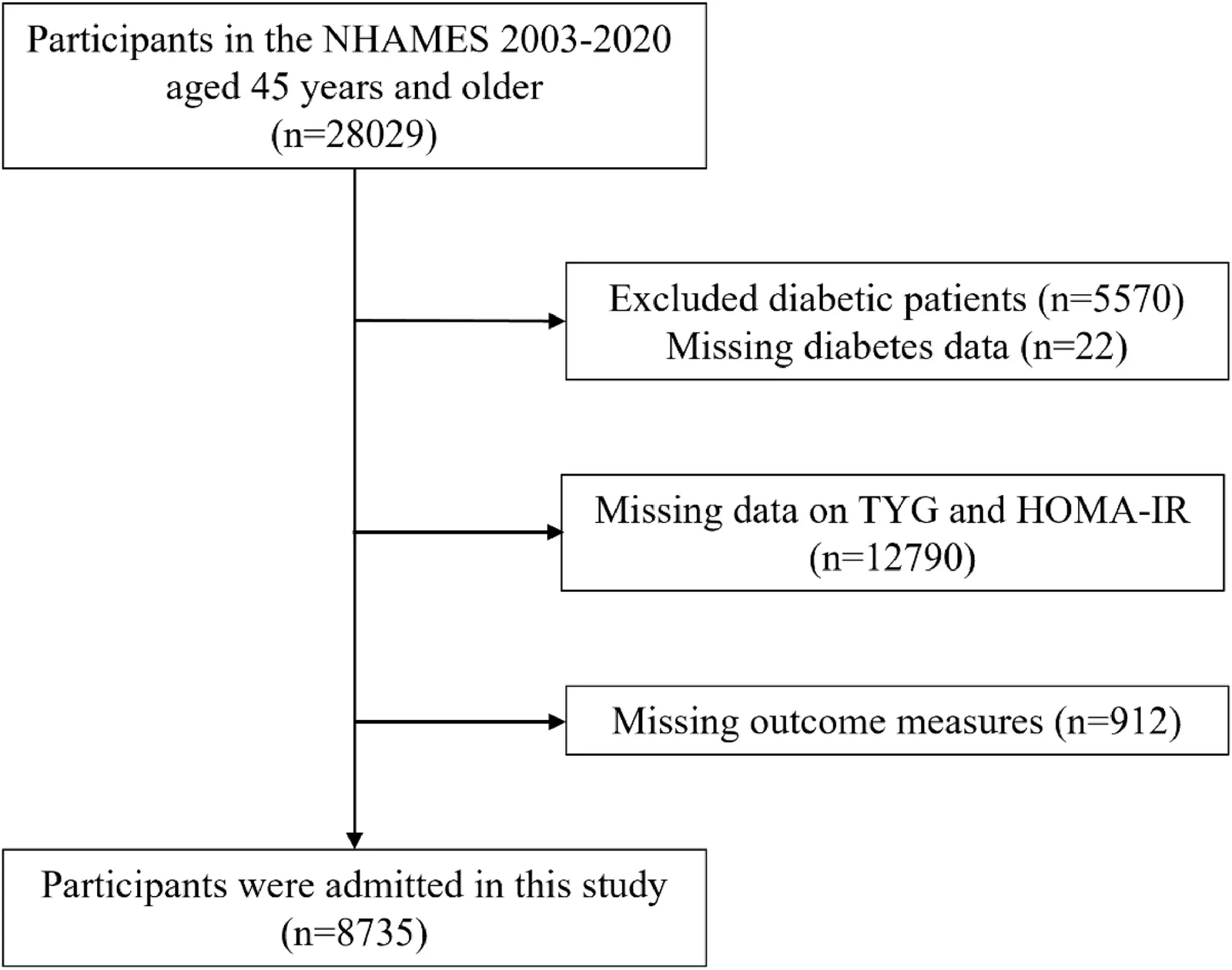
Flowchart of the included participants. TyG, Triglyceride-Glucose; HOMA-IR, homeostatic model assessment of insulin resistance.

Table 2 presents the associations of the TyG index and HOMA-IR with mortality outcomes based on Cox proportional hazards regression. When analyzed as a continuous variable, each unit increase in the TyG index was independently associated with elevated risks of all-cause mortality (Model 3 adjusted HR = 1.19, 95% CI: 1.06–1.34, p = 0.005), CVDs mortality (HR = 1.31, 95% CI: 1.05–1.64, p = 0.02), and cerebrovascular mortality (HR = 1.92, 95% CI: 1.33–2.77, p < 0.001). In tertile analyses, a graded positive association was observed only for cerebrovascular mortality, while for all‑cause mortality, significance persisted solely in the highest Tertile (T3) (HR = 1.26, 95% CI: 1.06–1.50, p = 0.01). No significant associations were found across TyG tertiles for CVDs or cardiac mortality. To further characterize the dose-response relationships, RCS analyses revealed that, after full adjustment, the TyG index exhibited approximately linear associations with all-cause and CVDs mortality (p for overall < 0.01, p for nonlinear > 0.05), but a significant nonlinear association with cerebrovascular mortality (Fig. 1).

**Table 2:** Association of the TyG and HOMA-IR index with All-cause mortality, CVDs/Cardiac/Cerebrovascular mortality.

Variable			All-cause mortality		CVDs mortality		Cardiac mortality		Cerebrovascular mortality	
			HR (95% CI)	p	HR (95% CI)	p	HR (95% CI)	p	HR (95% CI)	p
TyG										
Continuous	Model 1		1.15 (1.04, 1.27)	0.01	1.20 (1.01, 1.43)	0.05	1.18 (0.96, 1.45)	0.12	1.32 (1.03, 1.68)	0.03
	Model 2		1.14 (1.02, 1.27)	0.02	1.26 (1.02, 1.56)	0.04	1.22 (0.95, 1.57)	0.11	1.56 (1.13, 2.15)	0.01
	Model 3		1.19 (1.06, 1.34)	0.005	1.31 (1.05, 1.64)	0.02	1.25 (0.97, 1.62)	0.08	1.92 (1.33, 2.77)	<0.001
Tertiles										
T1			1.00 (Reference)							
T2	Model 1		1.19 (1.01, 1.39)	0.03	1.31 (0.97, 1.77)	0.08	1.21 (0.87, 1.69)	0.27	2.08 (1.08, 4.02)	0.03
	Model 2		1.11 (0.97, 1.27)	0.14	1.25 (0.93, 1.69)	0.13	1.15 (0.83, 1.60)	0.39	2.19 (1.11, 4.33)	0.02
	Model 3		1.13 (0.98, 1.30)	0.09	1.28 (0.95, 1.73)	0.10	1.17 (0.85, 1.63)	0.34	2.39 (1.25, 4.58)	0.01
T3	Model 1		1.25 (1.06, 1.47)	0.01	1.27 (0.93, 1.72)	0.18	1.18 (0.86, 1.63)	0.31	1.92 (1.05, 3.49)	0.03
	Model 2		1.20 (1.02, 1.41)	0.03	1.28 (0.93, 1.77)	0.13	1.18 (0.84, 1.66)	0.33	2.26 (1.19, 4.28)	0.01
	Model 3		1.26 (1.06, 1.50)	0.01	1.34 (0.97, 1.84)	0.07	1.20 (0.86, 1.67)	0.29	3.01 (1.56, 5.81)	0.001
HOMA-IR										
Continuous	Model 1		1.03 (1.01, 1.04)	<0.001	1.03 (1.01, 1.05)	0.002	1.03 (1.01, 1.05)	0.004	1.03 (0.99, 1.07)	0.13
	Model 2		1.02 (1.01, 1.03)	<0.001	1.02 (1.01, 1.04)	0.004	1.02 (1.01, 1.04)	0.01	1.02 (0.99, 1.05)	0.13
	Model 3		1.02 (1.01, 1.04)	<0.001	1.03 (1.01, 1.04)	0.004	1.02 (1.01, 1.05)	0.02	1.04 (1.02, 1.07)	<0.001
Tertiles										
T1			1.00 (Reference)							
T2	Model 1		0.99 (0.85, 1.16)	0.92	0.82 (0.61, 1.10)	0.19	0.75 (0.54, 1.03)	0.07	1.26 (0.73, 2.17)	0.41
	Model 2		0.92 (0.79, 1.06)	0.23	0.72 (0.55, 0.94)	0.02	0.65 (0.49, 0.87)	0.004	1.05 (0.60, 1.85)	0.86
	Model 3		0.93 (0.80, 1.08)	0.34	0.68 (0.52, 0.90)	0.01	0.60 (0.44, 0.82)	0.001	1.24 (0.70, 2.19)	0.46
T3		Model 1	1.15 (0.98, 1.35)	0.09	1.00 (0.71, 1.40)	0.10	1.01 (0.70, 1.45)	0.97	0.95 (0.55, 1.65)	0.87
		Model 2	1.13 (0.97, 1.30)	0.11	0.95 (0.69, 1.31)	0.75	0.96 (0.68, 1.36)	0.82	0.88 (0.51, 1.52)	0.65
		Model 3	1.13 (0.95, 1.35)	0.18	0.83 (0.58, 1.19)	0.31	0.78 (0.52, 1.16)	0.22	1.13 (0.62, 2.05)	0.69

TyG, Triglyceride-Glucose; HOMA-IR, Homeostatic Model Assessment of Insulin Resistance; CVDs, Cardiovascular and Cerebrovascular Diseases; T, Tertiles.

Model 1: Crude; Model 2: Adjust age, sex and race; Model 3: Adjust age, sex, race, smoking, alcohol, BMI, hypertension, dyslipidemia, prevalent cardiovascular diseases and stroke.

**Fig. 2:**
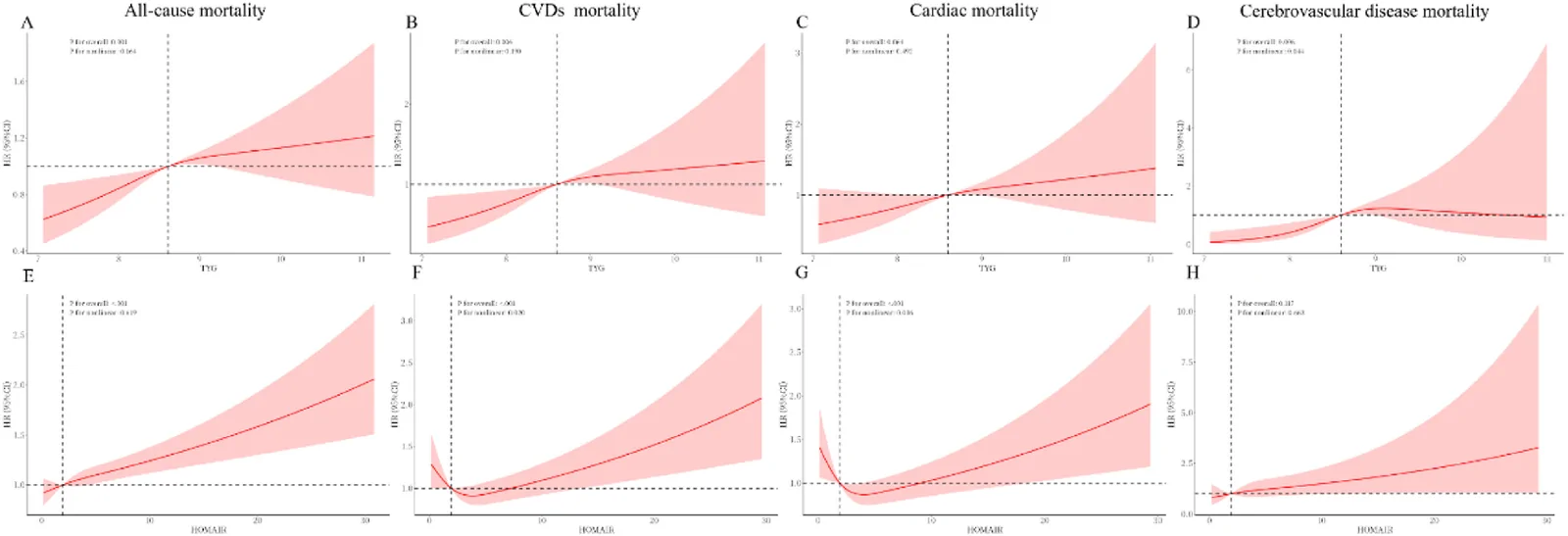
The RCS analysis between the TyG index and the All-cause mortality, CVDs/Cardiac/Cerebrovascular mortality. TyG, Triglyceride-Glucose; HOMA-IR, homeostatic model assessment of insulin resistance; CVDs, Cardiovascular and cerebrovascular diseases; RCS, restricted cubic spline.

When analyzed as a continuous variable, HOMA-IR showed significant linear associations with all-cause, CVDs and cardiac mortality, with each unit increase corresponding to a 2%–3% rise in risk in the fully adjusted model. Its association with cerebrovascular mortality became significant only after full adjustment (Model 3 HR = 1.04, 95% CI: 1.02–1.07, p < 0.001). However, both tertile-based and RCS analyses revealed more complex association patterns that differed by mortality endpoint. In tertile analysis, the middle Tertile (T2) was linked to a significantly lower risk of cardiac mortality compared with the lowest Tertile (T1) (Model 3 HR = 0.60, 95% CI: 0.44–0.82, p = 0.001), whereas the highest Tertile (T3) did not show a statistically elevated risk for any mortality outcome. RCS analyses further delineated these associations (Fig. 2), indicating a J-shaped relationship with CVDs and cardiac mortality, a linear association with all-cause mortality, and for cerebrovascular mortality, the continuous analysis showed a significant linear association (Table 2) while RCS revealed no significant departure from linearity (p for nonlinear > 0.05).

To assess the incremental predictive value for mortality outcomes, we compared the discrimination of models incorporating the TyG index or HOMA-IR against a base model (Model 3 covariates) using Harrell’s C-statistics (Table 3). For CVDs mortality, the C-statistics improved from 0.792 for the base model to 0.793 with TyG (ΔC = +0.001) and remained 0.792 with HOMA-IR. Similar minimal improvements were observed for cardiac and cerebrovascular mortality with TyG (ΔC = +0.001 and +0.002, respectively), whereas HOMA-IR showed negligible changes. These improvements in C-statistics were minimal and did not reach statistical significance, indicating that neither index significantly enhanced the overall discriminative ability of the base model for mortality prediction in this cohort.

**Table 3:** Comparison of model discrimination for mortality outcomes using C-index.

Model	C-index (95% CI)	Improvement (ΔC)	SE
CVDs mortality			
Base model	0.792 (0.771, 0.813)	Reference	0.0105
Base model + TyG index	0.793 (0.773, 0.814)	+0.001	0.0105
Base model + HOMA-IR	0.792 (0.771, 0.813)	-0.000	0.0106
Cardiac mortality			
Base model	0.789 (0.766, 0.813)	Reference	0.0119
Base model + TyG index	0.790 (0.767, 0.813)	+0.001	0.0119
Base model + HOMA-IR	0.789 (0.766, 0.813)	-0.000	0.0119
Cerebrovascular mortality			
Base model	0.812 (0.771, 0.853)	Reference	0.0209
Base model + TyG index	0.814 (0.772, 0.856)	+0.002	0.0214
Base model + HOMA-IR	0.811 (0.770, 0.853)	-0.001	0.0213

C-index, Concordance Index; TyG, Triglyceride-Glucose; HOMA-IR, Homeostatic Model Assessment of Insulin Resistance; CVDs, Cardiovascular and Cerebrovascular Diseases; SE, Standard Error.

### Associations with cause‑specific mortality under competing risks

Table 4 presents the results of the competing risk analysis for cause-specific mortality including CVDs, cardiac and cerebrovascular mortality. When accounting for deaths from other causes, the patterns of association for TyG and HOMA-IR differed notably from the traditional Cox models.

**Table 4:** Competing risk analysis of TyG index and HOMA-IR with mortality outcomes.

Variable		CVDs mortality		Cardiac mortality		Cerebrovascular mortality	
		HR (95% CI)	p	HR (95% CI)	p	HR (95% CI)	p
TyG							
T1		1.00 (Reference)					
T2	Model 1	1.35 (1.10, 1.66)	0.004	1.28 (1.02, 1.61)	0.03	1.63 (1.03, 2.59)	0.04
	Model 2	1.30 (1.06, 1.60)	0.01	1.25 (0.99, 1.57)	0.06	1.55 (0.97, 2.47)	0.07
	Model 3	1.32 (1.07, 1.62)	0.01	1.24 (0.99, 1.56)	0.07	1.65 (1.04, 2.63)	0.03
T3	Model 1	1.30 (1.06, 1.59)	0.01	1.26 (1.00, 1.58)	0.05	1.46 (0.91, 2.32)	0.11
	Model 2	1.31 (1.07, 1.62)	0.01	1.28 (1.02, 1.61)	0.04	1.44 (0.89, 2.33)	0.14
	Model 3	1.27 (1.02, 1.58)	0.03	1.20 (0.95, 1.53)	0.13	1.58 (0.95, 2.62)	0.08
HOMA-IR							
T1		1.00 (Reference)					
T2	Model 1	0.80 (0.66, 0.97)	0.02	0.74 (0.60, 0.93)	0.01	1.03 (0.68, 1.56)	0.89
	Model 2	0.82 (0.68, 1.00)	0.046	0.77 (0.62, 0.96)	0.02	1.07 (0.70, 1.62)	0.76
	Model 3	0.76 (0.62, 0.93)	0.01	0.69 (0.55, 0.87)	0.002	1.10 (0.72, 1.68)	0.66
T3	Model 1	0.82 (0.68, 0.99)	0.04	0.84 (0.68, 1.04)	0.10	0.76 (0.48, 1.19)	0.22
	Model 2	0.96 (0.79, 1.17)	0.68	0.98 (0.79, 1.22)	0.87	0.89 (0.56, 1.41)	0.62
	Model 3	0.79 (0.63, 0.98)	0.04	0.77 (0.60, 0.98)	0.03	0.94 (0.56, 1.59)	0.82

TyG, Triglyceride-Glucose; HOMA-IR, Homeostatic Model Assessment of Insulin Resistance; CVDs, Cardiovascular and Cerebrovascular Diseases; T, Tertiles.

Model 1: Crude; Model 2: Adjust age, sex and race; Model 3: Adjust age, sex, race, smoking, alcohol, BMI, hypertension, dyslipidemia, prevalent cardiovascular diseases and stroke.

In the competing risk framework, higher TyG index remained a significant predictor for CVDs mortality. Across the three adjustment models for the T2 and T3 groups, the HRs were consistently greater than 1, indicating a greater probability of dying from CVDs than from other causes. This remained significant in the fully adjusted model (Model 3: T2 HR = 1.32, p = 0.01; T3 HR = 1.27, p = 0.03). A similar but weaker trend was observed for cardiac mortality. For cerebrovascular mortality, the T2 group showed a significantly increased risk in Model 3 (HR = 1.65, 95% CI: 1.04–2.63, p = 0.03), whereas the T3 group exhibited an elevated but statistically non‑significant trend (HR = 1.58, 95% CI: 0.95–2.62, p = 0.08). In contrast, HOMA-IR showed an opposite pattern, with HRs below 1 for CVDs and cardiac mortality across models. Higher HOMA-IR (especially the middle tertile) was associated with a greater probability of dying from other causes than from CVDs or heart disease. No significant association was found for cerebrovascular mortality.

To examine the influence of pre-existing cardiovascular and cerebrovascular diseases, we performed a sensitivity analysis by excluding these individuals (Table 5). The results showed key differences from the main analysis that included all participants. For TyG, although the direction of association with CVDs and cardiac mortality was similar to the primary analysis (HRs 1.19–1.25), none reached statistical significance. Notably, in this subgroup, the highest Tertile (T3) showed a 102% increased risk of cerebrovascular mortality in the fully adjusted model (HR = 2.02, 95% CI: 1.08–3.75, p = 0.03), whereas the association for the middle Tertile (T2) was no longer significant (HR = 1.47, 95% CI: 0.81–2.66, p = 0.21). For HOMA-IR, after excluding participants with prevalent CVDs, the T3 group no longer showed a significantly lower relative probability of CVDs death compared with T1, whereas the HR < 1 in the T2 group remained significant.

**Table 5:** Sensitivity analysis excluding participants with prevalent CVDs: competing risk models for TyG index and HOMA-IR with mortality outcomes.

Variable		CVDs mortality		Cardiac mortality		Cerebrovascular mortality	
		HR (95% CI)	p	HR (95% CI)	p	HR (95% CI)	p
TyG							
T1		1.00 (Reference)					
T2	Model 1	1.25 (0.98, 1.60)	0.08	1.21 (0.92, 1.59)	0.17	1.43 (0.80, 2.56)	0.23
	Model 2	1.19 (0.93, 1.52)	0.18	1.16 (0.88, 1.52)	0.29	1.34 (0.74, 2.45)	0.34
	Model 3	1.19 (0.92, 1.52)	0.18	1.14 (0.86, 1.50)	0.37	1.47 (0.81, 2.66)	0.21
T3	Model 1	1.24 (0.97, 1.58)	0.09	1.12 (0.85, 1.48)	0.43	1.78 (1.02, 3.10)	0.04
	Model 2	1.24 (0.96, 1.59)	0.10	1.12 (0.85, 1.48)	0.43	1.77 (0.99, 3.17)	0.06
	Model 3	1.19 (0.92, 1.55)	0.19	1.03 (0.77, 1.38)	0.82	2.02 (1.08, 3.75)	0.03
HOMA-IR							
T1		1.00 (Reference)					
T2	Model 1	0.76 (0.60, 0.97)	0.03	0.70 (0.53, 0.92)	0.01	1.03 (0.62, 1.69)	0.92
	Model 2	0.77 (0.61, 0.99)	0.04	0.71 (0.54, 0.94)	0.02	1.05 (0.64, 1.74)	0.84
	Model 3	0.72 (0.57, 0.93)	0.01	0.64 (0.48, 0.85)	0.002	1.12 (0.67, 1.88)	0.67
T3	Model 1	0.88 (0.70, 1.11)	0.27	0.93 (0.72, 1.20)	0.55	0.70 (0.40, 1.23)	0.21
	Model 2	1.03 (0.81, 1.30)	0.84	1.08 (0.83, 1.40)	0.56	0.81 (0.46, 1.45)	0.48
	Model 3	0.87 (0.67, 1.14)	0.31	0.85 (0.63, 1.14)	0.28	0.93 (0.48, 1.79)	0.83

TyG, Triglyceride-Glucose; HOMA-IR, Homeostatic Model Assessment of Insulin Resistance; CVDs, Cardiovascular and Cerebrovascular Diseases; T, Tertiles.

Model 1: Crude; Model 2: Adjust age, sex and race; Model 3: Adjust age, sex, race, smoking, alcohol, BMI, hypertension, dyslipidemia.

## Discussion

We analyzed NHANES 2003–2020 data to assess TyG and HOMA-IR associations with mortality: all-cause, CVDs, cardiac and cerebrovascular disease. Results demonstrate that both the TyG index and HOMA-IR were associated with the risks of all-cause and CVDs mortality, yet their association patterns differed. The TyG index showed a steady positive relationship with CVDs mortality ‒ meaning a linear association across the full range of TyG values ‒ that persisted even after accounting for competing risks of other deaths. HOMA-IR, in contrast, exhibited a more complex and variable pattern. Although it showed significant linear associations as a continuous variable, its tertile and RCS analyses revealed a J-shaped nonlinear relationship specifically with CVDs and cardiac mortality, and did not provide as distinct a signal in the competing risk framework. Overall, the TyG index demonstrated a more consistent and interpretable linear association with CVDs mortality than HOMA-IR in this cohort.

Although the Euglycemic-Hyperinsulinemic Clamp (EHC) remains the gold standard for IR quantification, its procedural complexity restricts widespread clinical implementation.^20^ While HOMA-IR has been the historical surrogate of choice, its dependence on expensive insulin assays limits practicality.^21^ In contrast, the TyG index, requiring only triglycerides and glucose measurements, has emerged as a cost‑effective alternative^6^ and has shown diagnostic and prognostic advantages over HOMA-IR in certain cardiovascular contexts.^5^^,^^8^^,^^22^, ^23^, ^24^, ^25^ Extensive research in the NHANES database has established associations between the TyG index and cardiovascular mortality, yet significant population heterogeneity exists. Key nonlinear relationships emerge in specific cohorts: Zhang et al. identified a U-shaped association between the baseline TyG index with Cardiac and all-cause mortality in patients with diabetes or pre-diabetes,^26^ while Liu et al. observed a similar nonlinearity in younger individuals with diabetes.^27^ Given that hyperglycemia fundamentally alters TyG’s metabolic behavior, we focused on a non-diabetic population. In this cohort, our analysis revealed approximately linear associations of the TyG index with all-cause and CVDs mortality, contrasting with the nonlinear patterns reported in diabetic populations. For HOMA-IR, we observed a J-shaped relationship with CVDs and cardiac mortality, whereas its association with cerebrovascular mortality was linear. This pattern aligns with previous reports of complex, non-linear associations for HOMA-IR, while highlighting that the nature of these relationships may vary by specific cause of death.^28^

Nevertheless, prior studies have not accounted for competing risks, potentially obscuring the true predictive utility of TyG and HOMA-IR. To address this, we for the first time applied competing risk models to assess cause-specific mortality. The Fine-Gray subdistribution hazard model was selected because our primary interest was in predicting the cumulative incidence of cause-specific mortality in the presence of competing events ‒ a common scenario in elderly populations where non-cardiovascular deaths are frequent. This approach provides estimates relevant for risk prediction, though it differs from the cause-specific hazard model. However, it is important to recognize that while the Fine-Gray model is appropriate for predicting cause-specific mortality under competing risks, the resulting subdistribution hazard differs from the actual cause-specific hazard and warrants cautious interpretation. Consistent with the non-significant differences in predictive discrimination observed in traditional Cox models, the competing risk analysis revealed divergent association patterns. Specifically, whereas the TyG index consistently predicted a higher risk of CVDs mortality, HOMA-IR was associated with a lower subdistribution hazard, particularly for cardiac mortality. This suggests that, in the presence of competing risks, higher HOMA-IR levels may be disproportionately experiencing the competing event (i.e., non-CVDs mortality) before a cardiovascular death can occur. It is important to emphasize that this finding does not indicate a protective effect of moderate insulin resistance against cardiovascular death. Rather, it reflects that in the subdistribution hazard framework, individuals with higher HOMA-IR are more likely to experience competing events (non-CVDs mortality) before cardiovascular death can occur, which alters the relative hazard estimates. This pattern highlights the distinct risk profiles captured by the two markers rather than suggesting any causal protective effect. While we cannot rule out the influence of unmeasured or complex confounding, together, these analyses indicate that the two markers appear to reflect related but distinct biological or risk pathways, offering complementary insights rather than supporting a clear hierarchy.

In the sensitivity analysis that excluded individuals with pre-existing CVDs, the predictive ability of the TyG index was significantly attenuated and even disappeared compared with the main analysis. This suggests that its positive association with overall CVDs mortality largely depends on individuals with pre-existing disease at baseline. In contrast, the protective association of the middle Tertile (T2) of HOMA-IR with cardiac mortality remained robust even in this healthier subgroup, while the associations for its highest Tertile (T3) were no longer significant. This differential pattern reinforces that the two markers capture distinct risk profiles. In healthy populations, the TyG index showed limited short-term predictive power for overall mortality risk.^29^ However, among those with existing diseases, it may serve as an indicator for predicting mortality risk.^30^ Notably, in healthy individuals, a high TyG index (T3) emerged as an independent and strong risk factor for incident cerebrovascular mortality. This pattern suggests that the TyG index may be more relevant for predicting incident cerebrovascular events than for reflecting the progression of established cardiovascular disease,^9^^,^^31^ highlighting its potential utility in primary prevention settings. Building on this pattern, we hypothesize that TyG may be particularly relevant for identifying risk of incident cerebrovascular events, whereas its association with overall CVDs mortality may be partially mediated through progression of established disease. However, given the reduced sample size in this subgroup analysis, these findings should be considered hypothesis-generating and require confirmation in prospective studies.

The TyG index reflects IR through two core pathways: 1) Dysregulated fatty acid mobilization promotes hepatic very low density lipoprotein (VLDL) overproduction and elevated triglycerides;^32^ 2) Impaired insulin signaling reduces muscular/hepatic glucose uptake while increasing hepatic glucose output, raising fasting glucose.^33^ Thus, TyG quantifies both lipid and glucose dysregulation, which are central manifestations of IR.^34^ Its direct linkage to atherogenic dyslipidemia and hyperglycemia may explain its stronger and more consistent association with vascular event-related mortality.^35^ In contrast, HOMA-IR primarily estimates pancreatic insulin secretion and hepatic insulin sensitivity, capturing broader systemic metabolic dysregulation that may not translate directly into vascular risk. Therefore, the direct linkage of the TyG index to key atherogenic processes may explain its more consistent linear association with vascular event-related mortality observed in our study, whereas the broader metabolic dysregulation captured by HOMA-IR might underlie its more complex and nonlinear risk relationships.

There were several limitations in our study should be considered. First, substantially fewer cerebrovascular than cardiac deaths reduced statistical power for stratified analyses, particularly constraining cerebrovascular outcome precision. Second, C-statistic comparisons showed no significant improvement in predictive discrimination with either marker beyond traditional risk factors. The lack of improvement in model discrimination suggests that neither TyG nor HOMA-IR would be useful as population screening tools to identify high-risk individuals beyond traditional risk factors. Their value may lie instead in providing pathophysiological insights and identifying specific subgroups (e.g., cerebrovascular risk in healthy individuals) where targeted prevention might be considered. Third, the NHANES cohort's predominant U.S. composition limits generalizability to populations with distinct genetic, lifestyle, and dietary profiles. Third, reliance on single-baseline TyG measurements precludes evaluating longitudinal IR variations, potentially obscuring dynamic risk trajectories. Finally, the mechanistic interpretations for the superiority of TyG are hypothetical, as mediation analyses were not performed.

## Conclusion

Among non-diabetic older adults, elevated TyG index is associated with increased all-cause and cause-specific CVDs mortality. Within the analytical framework of this study, the TyG index showed a more consistent and interpretable linear association with CVDs mortality than HOMA-IR in this cohort. It may offer additional insight for cerebrovascular risk assessment. Given its accessibility and cost-effectiveness, future studies should investigate therapeutic interventions targeting TyG to improve survival in elderly patients.

## Availability of data and materials

All data used in this analysis were publicly available datasets from the NHANES. The data can be accessed at: https://www.cdc.gov/nchs/nhanes/.

## Ethics approval and consent to participate

The National Center for Health Statistics Ethics Review Board approved the NHANES protocols, and written informed consent was obtained from all participants.

## Authors’ contributions

Xintian Xu: Conceptualization, Formal analysis, Writing – original draft. Xiaomei Ye: Data curation. Yaozhuo Cai: Data curation. Chen Xiao: Formal analysis. Hao Chen: Writing – review & editing. Jingping Sun: Writing – review & editing. Xueli Cai: Funding acquisition. All authors have approved the final manuscript.

## Funding

This work was supported by grants from 10.13039/501100012165Zhejiang Provincial-Municipal Co-constructed Key Discipline in Neurology and the Key Science & Technologies R&D Program of Lishui City (2023ZDYF17).

## Consent for publication

Not applicable.

## Conflicts of interest

The authors declare no conflicts of interest.
